# Cigarette smoke induces genetic instability in airway epithelial cells by suppressing FANCD2 expression

**DOI:** 10.1038/sj.bjc.6604362

**Published:** 2008-05-13

**Authors:** L E Hays, D M Zodrow, J E Yates, M E Deffebach, D B Jacoby, S B Olson, J F Pankow, G C Bagby

**Affiliations:** 1 OHSU Cancer Institute, Oregon Health & Science University, 3181 Southwest Sam Jackson Park Road, Portland, OR 97239, USA; 2 Department of Medicine, Oregon Health & Science University, 3181 Southwest Sam Jackson Park Road, Portland, OR 97239, USA; 3 Veterans Administration Medical Center, 3710 Southwest United States Veteran's Hospital Road, Portland, OR 97239, USA; 4 Department of Molecular and Medical Genetics, Oregon Health & Science University, 3181 Southwest Sam Jackson Park Road, Portland, OR 97239, USA; 5 Department of Environmental and Biomolecular Systems, Oregon Graduate Institute, Oregon Health & Science University, 20000 Northwest Walker Road, Beaverton, OR 97006, USA

**Keywords:** lung cancer, carcinogenesis, tobacco, mechanisms of genomic alterations, Fanconi anaemia

## Abstract

Chromosomal abnormalities are commonly found in bronchogenic carcinoma cells, but the molecular causes of chromosomal instability (CIN) and their relationship to cigarette smoke has not been defined. Because the Fanconi anaemia (FA)/BRCA pathway is essential for maintenance of chromosomal stability, we tested the hypothesis that cigarette smoke suppresses that activity of this pathway. Here, we show that cigarette smoke condensate (CSC) inhibited translation of *FANCD2* mRNA (but not *FANCC* or *FANCG*) in normal airway epithelial cells and that this suppression of FANCD2 expression was sufficient to induce both genetic instability and programmed cell death in the exposed cell population. Cigarette smoke condensate also suppressed FANCD2 function and induced CIN in bronchogenic carcinoma cells, but these cells were resistant to CSC-induced apoptosis relative to normal airway epithelial cells. We, therefore, suggest that CSC exerts pressure on airway epithelial cells that results in selection and emergence of genetically unstable somatic mutant clones that may have lost the capacity to effectively execute an apoptotic programme. Carcinogen-mediated suppression of *FANCD2* gene expression provides a plausible molecular mechanism for CIN in bronchogenic carcinogenesis.

Lung cancer is the most common malignancy in the world causing over one million deaths per year (Kamangar *et al*, 2006). The major causative factor is cigarette smoke, which is linked to greater than 90% of cases (Jemal *et al*, 2001). Lung cancers display high levels of chromosomal instability (CIN) (Masuda and Takahashi, 2002), and preliminary evidence suggests that cigarette smoke condensate (CSC) may directly induce CIN (Luo *et al*, 2004). However, no specific biochemical or genetic mechanisms for this have been identified.

Potential carcinogenic effects of cigarette smoke can be modelled *in vitro* by exposing airway epithelial cells to CSC. This approach has been widely used to define biological responses to CSC (Andreoli *et al*, 2003), and, in some instances, to define specific categories of genetic damage induced by CSC (Albino *et al*, 2004; Luo *et al*, 2004). Few studies have clarified, however, the effects of CSC on caretaker pathways that protect the genome from environmental stressors. Because of the high incidence of aerodigestive malignancies in the inherited chromosomal instability disease Fanconi anaemia (FA), the universal presence of CIN in FA cells and complex cytogenetic defects in neoplastic FA cells, and the well-known biochemical role played by FA proteins in stabilising the genome (Bagby and Alter, 2006), we hypothesised that one or more of the chemicals in CSC might suppress the function of the FA pathway in normal individuals and destabilise the genome in exposed cells of the airway.

Fanconi anaemia is caused by biallelic (or X-chromosome-linked) inactivation of 1 out of 13 FA genes (Bagby and Alter, 2006; Dorsman *et al*, 2007; Smogorzewska *et al*, 2007), including *BRCA2* (*FANCD1*). Eight of the gene products form a nuclear core complex (FANCA, FANCB, FANCC, FANCE, FANCF, FANCG, FANCL, and FANCM), a complex that must be completely intact for FANCD2 to become monoubiquitinated. Therefore, to delineate the *in vitro* effects of CSC on FA gene expression in human airway epithelial cells, we measured formation of monoubiquitinated and nonubiquitinated FANCD2 after CSC treatment. Here, we show that CSC suppressed translation of *FANCD2* and that overexpression of FANCD2 protected cells from the toxic effects of cigarette smoke. The protective effects of FANCD2 were specific as CSC did not substantially decrease expression of other FA proteins (FANCC and FANCG) in airway epithelial cells and overexpression of these same FA proteins did not alter CSC-induced toxicity. Fancc-deficient cells, however, were hypersensitive to CSC demonstrating that it is the monoubiquitinated form of FANCD2 that protects cells against CSC. Furthermore, FANCD2 suppression induced CIN in exposed normal and neoplastic epithelial cells; however, bronchogenic carcinoma cells were resistant to CSC-induced toxicity.

## MATERIALS AND METHODS

### Cell culture and chemicals

**Figure 1:**
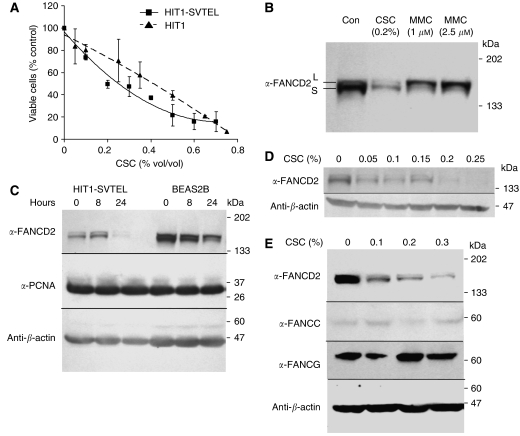
Cigarette smoke condensate downregulates FANCD2. (**A**) Survival analyses of primary (HIT1) and SV40- and hTERT-transformed (HIT1-SVTEL) human airway epithelial cells treated with indicated concentrations of CSC for 24 h are shown. Data points are means±s.d. from three experiments performed in triplicate. HIT1 and HIT1-SVTEL were not significantly different in their sensitivities to CSC (*P*=0.9, regression analysis). (**B**) Immunoblot of HIT1-SVTEL cells treated with indicated concentrations of CSC or MMC for 24 h (FANCD2-L=monoubiquitinated; FANCD2-S=nonubiquitinated) is shown. (**C**) Immunoblots of HIT1-SVTEL or BEAS2b (normal bronchial epithelial) cells treated with 0.2% CSC for indicated time intervals are shown. (**D**, **E**) Immunoblots of HIT1-SVTEL cells treated with indicated concentrations of CSC for 24 h are shown.

Primary airway epithelial cells were prepared from human tracheas obtained at Oregon Health & Science University (OHSU) using an Institutional Review Board-approved protocol by incubation with 0.1% pronase solution (Sigma-Aldrich, St Louis, Missouri, USA) and designated HIT1. A portion of the primary airway epithelial cells was transformed by retroviral transduction of SV40 large T-antigen (packaging line PA12/UL95 was derived from *ψ*2/UL95 provided by Dr Roger Cone at OHSU) and *hTERT* (packaging line PG13/hTERT kindly provided by Dr Denise Galloway at Fred Hutchinson Cancer Research Center) and designated HIT1-SVTEL. Transformed and untransformed primary airway epithelial cells displayed similar sensitivities to CSC (Figure 1A). HIT1-SVTEL cells ectopically expressing FANCD2, FANCC, or FANCG were derived by retroviral transduction and designated HIT1-SVTEL/FANCD2, HIT1-SVTEL/FANCC, or HIT1-SVTEL/FANCG, respectively. *FANCD2* retrovirus was produced from a pMMP vector as described previously (Garcia-Higuera *et al*, 2001). Retroviruses containing *FANCC* or *FANCG* were produced from 293T cells transfected with gag/pol, envelope, and MIEG3 plasmids containing *FANCC* or *FANCG* cDNA (all plasmids kindly provided by Dr Qishen Pang at Cincinnati Children's Hospital Medical Center). Murine tracheal airway epithelial cells and/or murine embryonic fibroblasts (MEFs) were prepared from wild-type, Fancd2-, and Fancc-deficient mice (kindly provided by Drs Markus Grompe at OHSU and Wade Clapp at Indiana University School of Medicine). A portion of the murine airway epithelial cells was transformed with SV40 (as described above). Murine embryonic fibroblasts were prepared from day 14 to 16 embryos. Bronchogenic cancer lines A549 and H292 were obtained from American Type Culture Collection. The *FANCD2*-mutant human fibroblast line PD20 and the isogenic *FANCD2*-complemented line PD20/D2 were obtained from the OHSU FA Cell Repository and Markus Grompe. Airway epithelial cells were grown in Dulbecco's Modified Eagle Medium (DMEM; Invitrogen, Carlsbad, CA, USA), whereas fibroblasts were grown in Minimal Essential Medium-alpha (both with 10% fetal bovine serum (FBS); Hyclone, Logan, UT, USA). Mitomycin C (MMC), epoxomicin, and cycloheximide (all from Sigma-Aldrich) were solubilised as per the manufacturer's instructions. Chromosomal breakage assays were performed as described previously (Pejovic *et al*, 2006).

### Preparation of cigarette smoke condensate

Mainstream CSC was sampled to collect both gas-phase and particle-phase constituents from the smoke generated from a major US brand of filtered, ‘full-flavour’ cigarette. Multiple samples were collected and utilised over the course of the project. For each, 20 cigarettes from a single pack were smoked according to the ‘Massachusetts’ smoking protocol (successive 45 ml puffs, each puff 2 s in duration, one puff every 30 s, cigarette smoke to within 23 mm of the filter overwrap paper; described in Hammond *et al*, 2006). As described elsewhere (Barsanti *et al*, 2007), the smoke passed through (a) 50 ml (70 cm long) chamber to allow particle coagulation; then (b) a jet orifice directed at the wall of a 20-ml glass vial (to collect particles). The flow was then directed to two bubble impingers each containing 5 ml of DMSO to collect gas-phase constituents. After all 20 cigarettes were smoked, the DMSO was used to wash the 50-ml chamber, then placed in a 20-ml vial. A total of 5 ml of additional DMSO was used to wash the impingers then added to the 20-ml vial. The vial was then sonicated for 30 s. After 4 h at room temperature in the dark, 6 ml of the clear supernatant was drawn off by pipette. Gentle volatilisation of an aliquot of the supernatant allowed the gravimetric determination of the total concentration of lower volatility constituents (e.g., tar) giving ∼25 mg ml^−1^. Each sample in DMSO was kept stored at 4°C until used for experiments.

### Survival and apoptosis assays

Cells were treated with different concentrations of CSC solubilised in DMSO for 8–24 h. In additional experiments, cells were treated with MMC for 24 h. Survival was measured with the dye-exclusion assay ViaCount (Guava Technologies, Hayward, CA, USA) and apoptosis was measured by either Annexin V (BD Biosciences, Bedford, MA, USA) or TUNEL staining (Guava Technologies). All assays were run and analysed with the Guava PCA-96 microcytometer.

### Immunoblotting

Cells were treated with CSC (0.05–0.25% which represents the amount of condensate extracted from roughly 1 to 5% of one cigarette) or MMC (1 and 2.5 *μ*M) for 24 h. Some were pretreated with epoxomicin for 2 h. Whole-cell lysates were prepared, separated by SDS-PAGE, and immunoblotted as described elsewhere (Pang *et al*, 2001). Primary antibodies used were mouse monoclonal anti-FANCD2 (diluted 1 : 200; Santa Cruz, Santa Cruz, CA, USA), rabbit polyclonal anti-*β*-actin (diluted 1 : 1000; Cell Signaling Technology, Beverly, MA, USA), mouse monoclonal anti-PCNA (1 : 2000; Cell Signaling Technology), rabbit polyclonals anti-FANCC, and anti-FANCG (1 : 1000; OHSU FA Antibody Project). Immunoblotting with anti-*β*-actin was performed on different aliquots of the same original samples immunoblotted with the different primary antibodies to confirm that total proteins levels were similar. Approximate molecular weights of FANCD-S, FANCD2-L, *β*-actin, PCNA, FANCC, and FANCG are 155, 162, 45, 36, 60, and 65 kDa, respectively. Secondary antibodies (1 : 10 000 dilution) were horseradish peroxidase-conjugated goat anti-mouse or goat anti-rabbit antibodies (Bio-Rad, Hercules, CA, USA). For FANCD2 half-life experiments, HIT1-SVTEL cells were treated simultaneously with 0.2% CSC and 20 *μ*g ml^−1^ cycloheximide (Sigma-Aldrich). Intensities of FANCD2 bands were determined by densitometry.

### RNA measurements

Total RNA was prepared from 5 × 10^6^ cells using the RNeasy Mini kit (Qiagen, Valencia, CA, USA). Complementary DNA synthesis and real-time PCR were performed as described previously (Pejovic *et al*, 2006). Predesigned primer and probe sets for *FANCD2* (Hs00696862_m1) and *PCNA* (Hs00945440_m1) were purchased as Taqman Gene Expression Assays from Applied Biosystems, and other primer/probe sets (Supplementary Table 1) were described previously (Pejovic *et al*, 2006).

### Protein synthesis experiments

HIT1-SVTEL cells were incubated in DMEM minus L-methionine and L-cysteine (Invitrogen) plus 10% dialysed FBS (Hyclone) for 30 min. For global protein synthesis experiments, cells were incubated with CSC for 1–2 h and then incubated with 50 *μ*Ci ml^−1^ [^35^S]-methionine/cysteine (PerkinElmer, Boston, MA, USA) for 30 min. [^35^S] incorporation was measured by liquid scintillation counting. For measurements of FANCD2 synthesis, HIT1-SVTEL, PD20, and PD20/FANCD2 cells were treated with either 0.2% CSC or DMSO plus approximately 300 *μ*Ci ml^−1^ [^35^S]-methionine/cysteine for 3 h. Whole-cell lysates were prepared as described above and then incubated consecutively with anti-FANCD2 monoclonal antibody and protein G-sepharose beads (Sigma-Aldrich). Washed pellets were then loaded onto 4–15% gradient acrylamide gels (Bio-Rad) and subjected to SDS-PAGE. FANCD2 was visualised by autoradiography. The intensities of FANCD2 bands were determined by densitometry.

### Densitometry

The relative intensities of FANCD2 bands were measured with Bio-Rad's Gel Documentation System and analysed with Quantity One software. The values of both FANCD2-L and FANCD2-S were combined for each lane/sample and treated samples were compared with controls.

### Statistics

*α*-Values for ANOVA (analysis of variance), linear regression analyses, and *t*-tests were 0.05. Analysis of variance test was two-way. The two-tailed one-sample *t*-test compared the mean of experimental results to the defined control value of 100%. Values shown in all graphs represent the means±s.d.

## RESULTS

To determine if CSC induced alterations in FA core complex function, we quantified FANCD2 protein in human airway epithelial cells at various times after exposure. Concentrations of CSC (0.05–0.25%) used represented approximately 1–5% of the yield from a single cigarette. The total amounts of FANCD2-S and FANCD2-L were markedly reduced after treatment (Figure 1B). However, both FANCD2-S and FANCD2-L were detectable, indicating that the eight core complex proteins were all functional. Maximal FANCD2 suppression occurred after 24 h of CSC exposure and occurred in a variety of airway epithelial cell lines (Figure 1C). FANCD2 suppression was detectable at doses as low as 0.05% CSC and was maximal at 0.2% (Figure 1D). The response of FANCD2 to CSC could not be attributed to any potential crosslinking agent in CSC, because it differed in these cells from the response induced by the crosslinking agent MMC. Mitomycin C, as expected (Garcia-Higuera *et al*, 2001), caused an almost complete conversion to FANCD2-L during the same treatment period (Figure 1B). The doses of MMC used in Figure 1B were approximately equitoxic to 0.05% CSC (Figure 1A and data not shown), and, as shown in Figure 1D, the effect of 0.05% CSC on FANCD2 expression was different from equitoxic doses of MMC (Figure 1B). We ruled out the possibility that CSC-induced FANCD2 reduction was due to mitotic arrest or cell growth inhibition by measuring both the level of the proliferation marker PCNA and the effects of CSC on cell-cycle distribution, neither of which changed at the 24 h time point (Figure 1C and data not shown). Furthermore, we examined whether CSC downregulated expression of other FA pathway members, although this seemed unlikely because FANCD2-L was still present in CSC-treated airway epithelial cells. As expected, neither FANCC nor FANCG expression was substantially downregulated after CSC treatment (Figure 1E).

**Figure 2:**
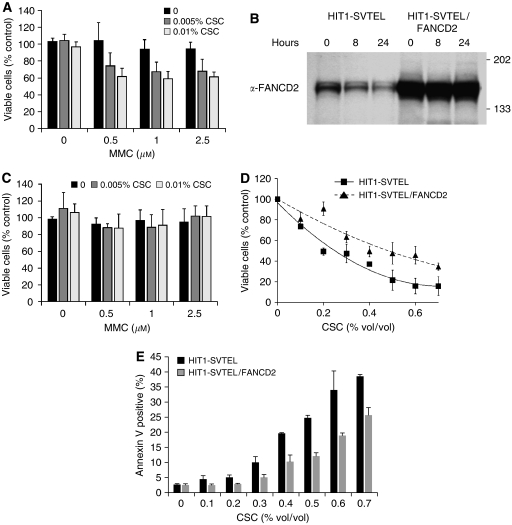
Decreased FANCD2 sensitises cells to CSC. (**A**) Survival analyses of HIT1-SVTEL cells treated sequentially with CSC and MMC for 24 h each (means±s.d. from three experiments performed in triplicate) are shown. Cigarette smoke condensate significantly sensitised HIT1-SVTEL cells to MMC at all concentrations (*P*<0.0001, ANOVA). (**B**) Immunoblot of HIT1-SVTEL cells with or without transduced *FANCD2* (HIT1-SVTEL/FANCD2=HIT1-SVTEL transduced with *FANCD2*) treated with 0.2% CSC for indicated time intervals are shown. (**C**) Survival analyses of HIT1-SVTEL/FANCD2 cells treated sequentially with CSC and MMC for 24 h each (means±s.d. from three experiments performed in triplicate) are shown. Cigarette smoke condensate did not sensitise HIT1-SVTEL/FANCD2 cells to MMC (*P*=0.9, ANOVA). (**D**) Survival analyses of HIT1-SVTEL and HIT1-SVTEL/FANCD2 cells treated with indicated concentrations of CSC for 24 h are shown. Data points are means±s.d. from three experiments performed in triplicate. HIT1-SVTEL/FANCD2 cells were more resistant than HIT1-SVTEL cells to CSC (*P*<0.0001, regression analysis). (**E**) Apoptosis measurements of HIT1-SVTEL and HIT1-SVTEL/FANCD2 cells treated with indicated concentrations CSC for 24 h are shown. Values are means±s.d. of triplicate samples in one of the three similar experiments.

Fanconi anaemia cells are so characteristically hypersensitive to crosslinking agents such as MMC that this forms the basis of the most widely utilised diagnostic test (Bagby and Alter, 2006). In nonneoplastic cells, the hypersensitivity is found both in survival and chromosomal breakage assays. To determine whether CSC-induced FANCD2 suppression was sufficient to induce the FA phenotype, we asked whether CSC sensitised cells to MMC. Treatment with CSC for 24 h significantly increased the sensitivity of airway epithelial cells to MMC in a survival assay (Figure 2A). FANCD2 suppression was sufficient to account for MMC hypersensitivity, because ectopic expression of FANCD2 protected cells from becoming MMC hypersensitive in response to CSC (Figures 2B and C).

**Figure 3:**
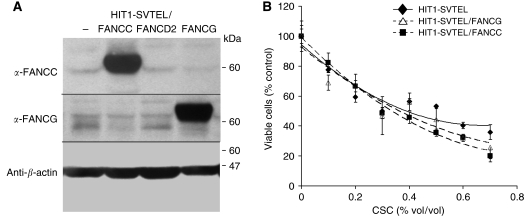
Ectopic expression of FANCC or FANCG does not affect CSC-induced cytotoxicity. (**A**) Immunoblots of HIT1-SVTEL cells (HIT1-SVTEL/−) transduced with either *FANCC* (HIT1-SVTEL/FANCC) or *FANCG* (HIT1-SVTEL/FANCG) are shown. (**B**) Survival analyses of HIT1-SVTEL, HIT1-SVTEL/FANCC, and HIT1-SVTEL/FANCG cells treated with indicated concentrations of CSC for 24 h are shown. Values represent means±s.d. of triplicate samples in one of the three similar experiments.

Toxicity of CSC is well documented (Andreoli *et al*, 2003). Because FA progenitor cells are hypersensitive to apoptotic cues (Lensch *et al*, 1999), we suspected that suppression of FANCD2 might be one cause of CSC-induced cell death. Indeed, CSC was toxic to airway epithelial cells, with an EC_50_ of approximately 0.3% vol/vol (Figure 2D) and a significant proportion of cell death was due to induction of apoptosis, as measured by Annexin V staining (Figure 2E). Overexpression of FANCD2 in airway epithelial cells increased the EC_50_ of CSC to 0.5% vol/vol (Figure 2D) and decreased the percentage of apoptotic cells after CSC treatment compared with HIT1-SVTEL cells (Figure 2E), confirming that FANCD2 suppression contributed in part to CSC-induced toxicity. To demonstrate that protective effects of FANCD2 were specific and not simply due to a consequence of overexpressing any FA/BRCA pathway member, we derived HIT1-SVTEL cells ectopically expressing either FANCC or FANCG (HIT1-SVTEL/FANCC or HIT1-SVTEL/FANCG, respectively; Figure 3A). In contrast to the protective effects of FANCD2, ectopic expression of neither FANCC nor FANCG changed the sensitivity of airway epithelial cells to CSC (Figure 3B).

**Figure 4:**
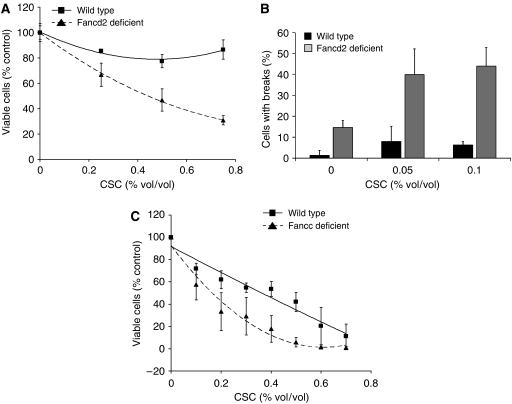
Fancd2- and Fancc-deficient cells are hypersensitive to CSC. (**A**) Survival analyses of primary wild-type and Fancd2-deficient murine airway epithelial cells treated with indicated concentrations of CSC for 24 h are shown. Values are means±s.d. of triplicate samples in one of the three similar experiments. Fancd2-deficient airway epithelial cells were significantly more sensitive to CSC than wild type (*P*<0.05, regression analysis). (**B**) Chromosomal breakage analyses of wild-type and Fancd2-deficient MEFs treated with indicated concentrations of CSC for 24 h are shown. Values represent means±s.d. from three independent cell lines. (**C**) Survival analyses of wild-type and Fancc-deficient transformed murine airway epithelial cells treated with indicated concentrations of CSC for 24 h are shown. Values are means±s.d. from three independent experiments performed in triplicate.

Using a genetic model to confirm that reduction/loss of FANCD2 sensitises cells to CSC, we tested susceptibility of airway epithelial cells and embryonic fibroblasts from Fancd2-deficient mice. As expected, Fancd2-deficient cells were hypersensitive to CSC in both cell survival (Figure 4A) and breakage assays (Figure 4B). In the breakage assays, low levels of CSC (0.05 and 0.1%) induced chromosomal breaks in 40–45% of Fancd2-deficient cells, but in only 5% of wild-type cells (Figure 4B). Fancc-deficient airway epithelial cells were also hypersensitive to CSC (Figure 4C), demonstrating that the presence of nonubiquitinated FANCD2 was not sufficient to provide protective effects against CSC-induced toxicity.

**Figure 5:**
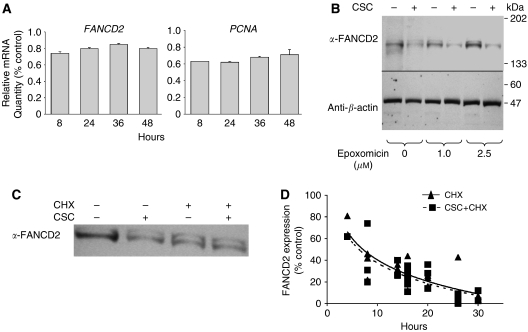
Cigarette smoke condensate does not inhibit transcription of *FANCD2* or degradation of FANCD2. (**A**) *FANCD2* and *PCNA* mRNA levels after exposure to 0.2% CSC for indicated times (means±s.d. from a representative experiment of two performed in triplicate) are shown. Values are relative to controls (0 h time point). (**B**) Immunoblots of HIT1-SVTEL cells treated where indicated with different concentrations of epoxomicin for 2 h and then 0.2% CSC for 24 h are shown. (**C**) Immunoblot of HIT1-SVTEL cells treated with 0.2% CSC and/or 20 *μ*g ml^−1^ cycloheximide (CHX) for 16 h is shown. (**D**) FANCD2 measured densitometrically from three experiments of HIT1-SVTEL cells treated with 0.2% CSC and/or 20 *μ*g ml^−1^ CHX for indicated hours (mean±s.d.) is shown. Solid and dashed lines represent cells treated with CHX alone or CHX and CSC, respectively.

Reduced FANCD2 did not correlate with reduced proliferation or growth of treated cells (Figure 1C). To determine general mechanisms by which FANCD2 expression was suppressed, we measured *FANCD2* and *PCNA* mRNA levels after CSC treatment using quantitative real-time PCR (Figure 5A). After 8–24 h of treatment, *FANCD2* RNA declined slightly; a decline that could not account for the roughly 90% decrease in FANCD2 protein by 24 h (Figures 1B and C). Cigarette smoke condensate exposure did not significantly alter transcripts of 27 other FA or DNA repair genes (Supplementary Table 1).

Reasoning that decline of FANCD2 in exposed cells likely reflected either enhanced proteolysis, suppression of translation, or both, we tested both possibilities. We first treated cells in advance of CSC treatment with a proteasome inhibitor (epoxomicin). Treatment had little or no effect on FANCD2 levels alone and did not reverse CSC-induced suppression (Figure 5B). We also quantified the effects of CSC on the degradation rate of FANCD2. Using a dose of cycloheximide that inhibited global RNA translation by 95% after 3 h (data not shown), the half-life of FANCD2 (approximately 8 h) was not altered by cotreatment with 0.2% CSC (Figures 5C and D).

**Figure 6:**
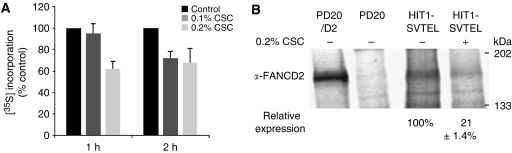
Cigarette smoke condensate inhibits *FANCD2* translation. (**A**) Cigarette smoke condensate significantly inhibited [^35^S] incorporation at 2 h for both concentrations (means±s.d. from three experiments performed in duplicate, *P*=0.009, ANOVA). (**B**) Autoradiogram of immunoprecipitated [^35^S]-FANCD2 in indicated cells treated with or without CSC for 3 h is shown. PD20/D2 and PD20 cells are positive and negative controls, respectively. FANCD2 expression was measured densitometrically and relative expression was calculated in CSC-treated HIT1-SVTEL cells by comparison with untreated cells (set to 100%). Values represent the mean and standard deviation from three independent experiments. FANCD2 synthesis in HIT1-SVTEL cells was significantly lower after CSC treatment (*P*=0.005, *t*-test).

It is known that cigarette smoke inhibits global protein translation *in vitro* and *in vivo* in the airway (Yeager, 1969; Hamosh *et al*, 1979), but there is no known linkage of that general effect with a specific mechanism of carcinogenesis. We confirmed global translational effects of CSC by measuring differences in [^35^S]-methionine/cysteine incorporation after CSC treatment. Within 2 h of exposure, CSC significantly inhibited translation (Figure 6A). To confirm that translation of *FANCD2* mRNA was suppressed, we measured [^35^S]-methionine/cysteine incorporation into FANCD2 protein by immunoprecipitation (Figure 6B). Within 3 h of exposure to CSC, [^35^S]-methionine/cysteine FANCD2 levels were reduced by approximately 80% compared with untreated controls. Therefore, global translation is suppressed and *FANCD2* mRNA is not exempt. That suppression of this particular transcript is of relevance to carcinogenesis and CIN is confirmed by the protective effect of ectopic FANCD2 expression (Figures 2C–E).

**Figure 7:**
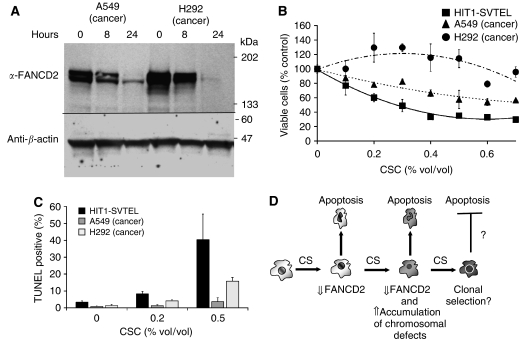
Bronchogenic carcinoma cells are resistant to CSC-induced toxicity but not FANCD2 downregulation. (**A**) Immunoblots of bronchogenic carcinoma cells (A549 and H292) treated for 8 and 24 h with 0.2% CSC are shown. (**B**) Survival analyses of cells treated for 24 h (means±s.d. from a representative of three independently performed experiments in triplicate) with indicated concentrations of CSC are shown. Cancer lines A549 and H292 showed different responses to treatment with CSC than HIT1-SVTEL (*P*=0.02, regression analyses). (**C**) Apoptotic responses in cells treated for 24 h (means±s.d. from a representative of two experiments performed in triplicate) with indicated concentrations of CSC are shown. (**D**) A proposed model of CSC-induced CIN is shown. Exposure of cigarette smoke (CS) downregulates FANCD2 expression and induces apoptosis in a proportion of exposed cells. Re-exposure to CS induces chromosomal damage due to decreased FANCD2 and facilitates selection of clones that may become resistance to the apoptosis-inducing actions of CS.

To determine if neoplastic cells retained responsiveness to CSC, we measured the effects of CSC on FANCD2 expression in bronchogenic carcinoma cells (A549 and H292). Cigarette smoke condensate suppressed FANCD2 in cancer cells (Figure 7A), but these cells were significantly more resistant to CSC in survival (Figure 7B) and apoptosis assays (Figure 7C) than were normal airway epithelial cells. Thus, bronchogenic carcinoma cells (A549 and H292) do exhibit CSC-induced FANCD2 suppression, but have become partially resistant to CSC.

## DISCUSSION

We quantified native and monoubiquitinated FANCD2 in airway epithelial cells to screen for dysfunction of the FA/BRCA pathway. We found that CSC suppressed translation of *FANCD2* mRNA, reducing both forms of FANCD2 and sensitising epithelial cells of the airway to DNA crosslinking agents. Linkage of the cytotoxic effects of CSC with FANCD2 suppression was confirmed in murine Fancd2-deficient cells, which were found to be sensitive to both CSC-induced toxicity and chromosomal breakage. In gain-of-function studies, overexpression of FANCD2, but not FANCC or FANCG, in normal airway epithelial cells protected CSC-exposed cells from undergoing apoptosis. Finally, CSC downregulated FANCD2 in bronchogenic carcinoma cells, but these cells were partially resistant to CSC-induced toxicity.

Although the correlation between lung cancer, cigarette smoke, and CIN has been well established (Jemal *et al*, 2001; Masuda and Takahashi, 2002), the molecular mechanisms by which cigarette smoke induces chromosomal damage have not been well defined. Here, we identify CSC-induced suppression of *FANCD2* gene expression as a novel mechanism of cigarette smoke-induced CIN and demonstrate for the first time carcinogen-induced repression of the FA/BRCA pathway. It is widely recognised that FA proteins promote chromosomal stability, FA cells display both endogenous and DNA crosslinker-induced CIN, and monoubiquitination of FANCD2 plays key regulatory roles (reviewed in Bagby and Alter, 2006). However, the precise biochemical mechanisms by which these molecules control chromosomal stability have not been delineated. Whether the role of the proteins is direct or indirect is unclear. Several lines of evidence suggest FA proteins are involved in DNA repair and recombination (Mathew, 2006), including the identification of *FANCD1* as *BRCA2* (Howlett *et al*, 2002). More recently, two FA genes, *FANCJ* and *FANCM*, were found to possess DNA helicase and/or endonuclease motifs (Levitus *et al*, 2005; Litman *et al*, 2005; Meetei *et al*, 2005). Other roles of FA proteins in preventing CIN have been suggested, including prevention of oxidative damage induced by reactive oxygen species or cytokine exposure (Pagano, 2000; Cumming *et al*, 2001; Zhang *et al*, 2007).

Analysis of the Fanconi Anemia Transcriptome Consortium Public Release (http://www.genesifter.net/web/hematology1a.html), a publicly available gene expression array database comparing normal and FA bone marrow RNA, reveals potential noncanonical roles of FA proteins in mediating chromosomal stability. Specifically, 2035 RNA transcripts are significantly decreased in FA bone marrow compared with normal controls (0.75-fold or less with a *P*-value of 0.05 corrected for multiple comparisons using Benjamini and Hochberg method), and, of these transcripts, the largest significant ontological categories include mitotic cell cycle, spindle organisation and biogenesis, and several related categories (see Supplementary Tables 2 and 3 for lists of individual transcripts). Thus, FA proteins may mediate chromosomal stability by regulating the expression of genes that stabilise the mitotic machinery.

Cigarette smoke-induced inhibition of global protein synthesis has been demonstrated by several groups in different tissues/cell types (lung, alveolar macrophages, and liver), and in both *in vitro* and *in vivo* animal models (Yeager, 1969; Garrett and Jackson, 1979; Hamosh *et al*, 1979; Leffingwell and Low, 1979; Hellstern *et al*, 1990). The mechanism of CSC-induced protein synthesis inhibition remains enigmatic. Of relevance to the studies herein, the linkage of translational suppression with suppression of specific proteins that mediate CSC-induced toxicity and chromosomal damage has not been previously reported. Here, we confirmed that CSC inhibited global protein synthesis *in vitro* in our model system of lung epithelial cells and identified downregulation of FANCD2, clearly indicating that *in vivo* studies are warranted.

Because translation is globally suppressed, any single or combination of proteins could theoretically contribute to CSC-induced CIN. However, while we cannot rule out the possibility that other downregulated proteins contribute to this response, we did confirm directly the significance of FANCD2 in mediating chromosomal stability by ectopically expressing FANCD2 in epithelial cells and demonstrating a protective effect against CSC-induced toxicity (Figure 2D). FANCD2 overexpression also protected cells against MMC-induced sensitivity in CSC-exposed cells (Figure 2C). Furthermore, CSC did not substantially affect the expression of FA proteins FANCC and FANCG (Figure 1E) and ectopic expression of these FA proteins did not provide protective effects against CSC (Figure 3B). Thus, downregulation of FANCD2 appears to be a critical event in cigarette smoke-induced CIN.

Fanconi anaemia cells are hypersensitive to a variety of apoptotic cues (Rosselli, 1998) and oxidative stress (Pagano and Youssoufian, 2003), and there is emerging evidence that for neoplastic clones to evolve their progenitors must adapt by inactivating precisely the apoptotic signalling pathways aberrantly activated as a result of FA gene inactivation (Lensch *et al*, 1999). Whether somatic events are adaptive or arise by selection of pre-existing mutant clones is not yet known, but the process is likely facilitated by the inherent genetic instability of FA cells. This theoretical model of carcinogenesis is perfectly congruent with observations here that CSC suppresses FANCD2 levels and, as a consequence, induces both genetic instability and apoptosis. Furthermore, we found that bronchogenic carcinoma cells were still sensitive to CSC-induced FANCD2 downregulation and thus CIN, but were at least in part resistant to CSC-induced apoptosis. Owing to well-established variations in survival responses of *in vitro* cell lines, we cannot conclusively demonstrate that cancer cells are more resistant to CSC than nonneoplastic epithelial cells of the airway. However, another group recently reported similar results (Kode *et al*, 2006). In this study, three lung cancer cell lines, only one of which was the same as used in our study (A549), displayed less cytotoxicity in response to cigarette smoke extract than primary small airway epithelial cells.

We, therefore, propose a model of the role of FANCD2 suppression in cigarette smoke-induced lung cancer (Figure 7D) in which cigarette smoke suppresses FANCD2 *in vivo*, creating recurring cycles of cytotoxicity and CIN. Suppression of *FANCD2* translation renders cells more sensitive to clastogen/DNA-damaging agents present in cigarette smoke upon re-exposure to cigarette smoke. These responses likely create perfect conditions for the selection of new clones that resist apoptotic cues including those contained in cigarette smoke.
