# Supplementary material for: Cigarette smoke induces genetic instability in airway epithelial cells by suppressing FANCD2 expression
Source: Br J Cancer. 2008 May 13;98(10):1653–61. doi: 10.1038/sj.bjc.6604362 (PMC2391131; doi:10.1038/sj.bjc.6604362)
Supplement: Supplementary Table 1 [file 6604362x1.doc]

Supplementary Table 1: Relative mRNA levels of DNA repair and FA genes, after 0.2% CSC exposure for 24 hrs.

| GeneSymbol | Gene Name | Expt 1Fold Change | Expt 2Fold Change |
| --- | --- | --- | --- |
| ATR | ataxia telangiectasia, Rad3-related | -1.1 (-1.0 to -1.1) | 1.2 (1.2-1.3) |
| **BID** | BH3 interacting domain death agonist | -2.4 (-2.4 to -2.5) | 1.4 (1.3-1.4) |
| **BLM** | Bloom syndrome | -1.4 (-1.3 to -1.6) | 1.5 (1.5-1.6) |
| **BRCA1** | breast cancer 1, early onset | -1.8 (-1.5 to -2.0) | 1.4 (1.3-1.5) |
| **BRCA2** | breast cancer 2, early onset | -1.7 (-1.6 to -1.8) | 1.3 (1.2-1.4) |
| **DCLRE1C** | DNA crosslink repair 1C (PSO2 homolog, S. cerevisiae) | -1.6 (-1.4 to -1.9) | 1.2 (1.2-1.2) |
| **ERCC1** | excision repair cross-complementing rodent repair deficiency 1 | -1.7 (-1.6 to -1.8) | 1.1 (1.0-1.1) |
| **ERCC4** | excision repair cross-complementing rodent repair deficiency 2 | -1.2 -1.2 to -1.3) | 1.4 (1.3-1.4) |
| **H2AFX** | H2A histone family, member X | -2.2 (-2.1 to -2.2) | 1.0 (1.0-1.1) |
| **HTATIP** | HIV-1 Tat interactive protein, 60kDa | -1.5 (-1.5 to -1.6) | -1.0 (-1.0 to -1.1) |
| **MRE11A** | mitotic recombination 11 | -1.6 (-1.5 to -1.7) | 1.0 (1.0-1.1) |
| **NBN** | nibrin, p95 protein of MRE11/RAD50 complex | -2.6 (-2.4 to -2.7) | -1.4 (-1.3 to -1.5) |
| **PCNA** | proliferating cell nuclear antigen | -1.6 (-1.5 to -1.6) | 2.0 (1.9-2.0) |
| **RAD51** | RAD51 homolog | -1.4 (-1.3 to -1.4) | 1.2 (1.2-1.2) |
| RAD54L | RAD54-like (S. cerevisiae) | -1.6 (-1.5 to -1.8) | -1.1 (-1.0 to -1.1) |
| **REV3L** | REV3-like, catalytic subunit of DNA polymerase zeta (yeast) | -2.0 (-1.7 to -2.3) | 1.5 (1.4-1.6) |
| **XRCC2** | X-ray repair complementing defective repair in CHO cells 2 | -1.6 (-1.6 to -1.7) | 1.3 (1.2-1.3) |
| **XRCC3** | X-ray repair complementing defective repair in CHO cells 3 | -1.9 (-1.9 to -2.0) | 1.0 (1.0-1.0) |
| **FANCA** | FA complementation group A | -1.5 (-1.5 to -1.6) | -1.1 (-1.0 to -1.1) |
| **FANCB** | FA complementation group B | -1.4 (-1.2 to -1.5) | 1.4 (1.3-1.5) |
| **FANCC** | FA complementation group C | -1.7 (-1.6 to -1.8) | -1.1 (-1.1 to -1.1) |
| **FANCD2** | FA complementation group D2 | 1.3 (1.2 to 1.3) | -1.2 (-1.2 to -1.3) |
| **FANCE** | FA complementation group E | 1.0 (1.0-1.1) | 1.3 (1.2-1.4) |
| **FANCF** | FA complementation group F | -1.1 (-1.0 to -1.1) | -1.1 (-1.0 to -1.2) |
| **FANCG** | FA complementation group G | -1.6 (-1.4 to -1.7) | 1.2 (1.1-1.2) |
| **FANCJ** | FA complementation group J | -2.0 (-1.9 to -2.1) | -1.5 (-1.4 to -1.5) |
| **FANCL** | FA complementation group L | -1.7 (-1.7 to -1.8) | 1.0 (1.0-1.0) |
| **FANCM** | FA complementation group M | 1.0 (0.9-1.1) | 1.4 (1.3-1.5) |
| **FANCN** | FA complementation group N | -1.4(-1.4 to –1.5) | 1.3 (1.2-1.3) |

Fold Change is expressed as a mean value, with the range of values measured in parentheses.
