# Supplementary material for: Cigarette smoke induces genetic instability in airway epithelial cells by suppressing FANCD2 expression
Source: Br J Cancer. 2008 May 13;98(10):1653–61. doi: 10.1038/sj.bjc.6604362 (PMC2391131; doi:10.1038/sj.bjc.6604362)
Supplement: Supplementary Table 2 [file 6604362x2.doc]

Supplementary Table 2: Mitotic Cell Cycle Genes Down-regulated in FA Bone Marrow.

| **Gene Identifier** | **Gene Title** |
| --- | --- |
| NM_004523 | Kinesin family member 11 |
| NM_001211 | BUB1 budding uninhibited by benzimidazoles 1 homolog beta (yeast) |
| BC005383 | Centrin, EF-hand protein, 3 (CDC31 homolog, yeast) |
| NM_004642 | CDK2-associated protein 1 |
| NM_020242 | Kinesin family member 15 |
| NM_016359 | Nucleolar and spindle associated protein 1 |
| BG105365 | S-phase kinase-associated protein 2 (p45) |
| NM_002358 | MAD2 mitotic arrest deficient-like 1 (yeast) |
| AF020043 | Structural maintenance of chromosomes 3 |
| AL353950 | Protein phosphatase 3 (formerly 2B), catalytic subunit, alpha isoform |
| NM_018123 | asp (abnormal spindle) homolog, microcephaly associated (Drosophila) |
| NM_006444 | Structural maintenance of chromosomes 2 |
| M13436 | Inhibin, beta A (activin A, activin AB alpha polypeptide) |
| NM_003981 | Protein regulator of cytokinesis 1 |
| NM_002485 | Nibrin |
| NM_007057 | ZW10 interactor |
| NM_007375 | TAR DNA binding protein |
| D80000 | Structural maintenance of chromosomes 1A |
| NM_004354 | Cyclin G2 |
| NM_001706 | B-cell CLL/lymphoma 6 (zinc finger protein 51) |
| D88357 | Cell division cycle 2, G1 to S and G2 to M |
| NM_002266 | Karyopherin alpha 2 (RAG cohort 1, importin alpha 1) |
| AL136877 | Structural maintenance of chromosomes 4 |
| BF112006 | RAN, member RAS oncogene family |
| NM_005563 | Stathmin 1/oncoprotein 18 |
| AW134535 | Cyclin G2 |
| NM_018131 | Centrosomal protein 55kDa |
| NM_003318 | TTK protein kinase |
| NM_014750 | Discs, large homolog 7 (Drosophila) |
| BC005978 | Karyopherin alpha 2 (RAG cohort 1, importin alpha 1) |
| AL524035 | Cell division cycle 2, G1 to S and G2 to M |
| NM_000919 | Peptidylglycine alpha-amidating monooxygenase |
| NM_004661 | Cell division cycle 23 homolog (S. cerevisiae) |
| X62048 | WEE1 homolog (S. pombe) |
| AF043294 | BUB1 budding uninhibited by benzimidazoles 1 homolog (yeast) |
| NM_006101 | NDC80 homolog, kinetochore complex component (S. cerevisiae) |
| AI346350 | Cyclin A2 |
| NM_001786 | Cell division cycle 2, G1 to S and G2 to M |
| NM_001255 | Cell division cycle 20 homolog (S. cerevisiae) |
| NM_003158 | aurora kinase A |
| AF054183 | RAN, member RAS oncogene family |
| AA911231 | Protein phosphatase 3 (formerly 2B), catalytic subunit, alpha isoform |
| AI796269 | Nibrin |
| NM_017760 | Non-SMC condensin II complex, subunit G2 |
| NM_017489 | Telomeric repeat binding factor (NIMA-interacting) 1 |
| NM_003157 | NIMA (never in mitosis gene a)-related kinase 4 |
| NM_007019 | Ubiquitin-conjugating enzyme E2C |
| NM_000075 | Cyclin-dependent kinase 4 |
| AK022511 | Non-SMC condensin I complex, subunit D2 |
| M68520 | Cyclin-dependent kinase 2 |
| AF213033 | Cyclin-dependent kinase inhibitor 3 (CDK2-associated dual specificity phosphatase) |
| NM_005496 | Structural maintenance of chromosomes 4 |
| BF435809 | Cullin 5 |
| NM_004701 | Cyclin B2 |
| BE407516 | Cyclin B1 |
| NM_022346 | Non-SMC condensin I complex, subunit G |
| AK026678 | Stromal antigen 2 |
| AF098158 | TPX2, microtubule-associated, homolog (Xenopus laevis) |
| NM_022346 | Non-SMC condensin I complex, subunit G |
| NM_005263 | Growth factor independent 1 |
| NM_000430 | Platelet-activating factor acetylhydrolase, isoform Ib, alpha subunit 45kDa |
| AI022882 | Peptidylglycine alpha-amidating monooxygenase |
| NM_001432 | Epiregulin |
| AF062537 | Cullin 3 |
| NM_012177 | F-box protein 5 |
| NM_004087 | Discs, large homolog 1 (Drosophila) |
| BC000229 | MIS12, MIND kinetochore complex component, homolog (yeast) |
| NM_006265 | RAD21 homolog (S. pombe) |
| BC001081 | Anaphase promoting complex subunit 5 |
| NM_003503 | Cell division cycle 7 homolog (S. cerevisiae) |
| U77949 | Cell division cycle 6 homolog (S. cerevisiae) |
| NM_014708 | Kinetochore associated 1 |
| NM_002497 | NIMA (never in mitosis gene a)-related kinase 2 |
| AI633566 | Microtubule-associated protein, RP/EB family, member 1 |
| T33068 | Anaphase promoting complex subunit 5 |
| AJ297710 | Cell division cycle 2-like 5 (cholinesterase-related cell division controller) |
| NM_001168 | Baculoviral IAP repeat-containing 5 (survivin) |
| U63743 | Kinesin family member 2C |
| NM_012325 | Microtubule-associated protein, RP/EB family, member 1 |
| AI347136 | Telomeric repeat binding factor (NIMA-interacting) 1 |
