# Supplementary material for: Cigarette smoke induces genetic instability in airway epithelial cells by suppressing FANCD2 expression
Source: Br J Cancer. 2008 May 13;98(10):1653–61. doi: 10.1038/sj.bjc.6604362 (PMC2391131; doi:10.1038/sj.bjc.6604362)
Supplement: Supplementary Table 3 [file 6604362x3.doc]

Supplementary Table 3: Spindle Organization and Biogenesis Genes Down-Regulated in FA Bone Marrow.

| **Gene Identifier** | **Gene Title** |
| --- | --- |
| NM_004523 | Kinesin family member 11 |
| NM_001211 | BUB1 budding uninhibited by benzimidazoles 1 homolog beta (yeast) |
| NM_001827 | CDC28 protein kinase regulatory subunit 2 |
| AF020043 | Structural maintenance of chromosomes 3 |
| NM_003981 | Protein regulator of cytokinesis 1 |
| NM_007057 | ZW10 interactor |
| D80000 | Structural maintenance of chromosomes 1A |
| BF112006 | RAN, member RAS oncogene family |
| NM_005563 | Stathmin 1/oncoprotein 18 |
| NM_003318 | TTK protein kinase |
| NM_006101 | NDC80 homolog, kinetochore complex component (S. cerevisiae) |
| NM_003158 | aurora kinase A |
| AF054183 | RAN, member RAS oncogene family |
| NM_007019 | Ubiquitin-conjugating enzyme E2C |
